# IMpower, CASPIAN, and more: exploring the optimal first-line immunotherapy for extensive-stage small cell lung cancer

**DOI:** 10.1186/s13045-020-00898-y

**Published:** 2020-06-05

**Authors:** Chengliang Huang, Gregory N. Gan, Jun Zhang

**Affiliations:** 1 Department of Respiratory and Critical Care Medicine II, The Affiliated Hospital of Southwest Medical University, 25 Taiping Street, Luzhou, 646000 Sichuan China; 2 Division of Medical Oncology, Department of Internal Medicine, University of Kansas Cancer Center, University of Kansas Medical Center, 3005 Wahl Hall East, 3901 Rainbow Blvd, Kansas City, KS 66160 USA; 3 Department of Radiation Oncology, University of Kansas Cancer Center, University of Kansas Medical Center, 3005 Wahl Hall East, 3901 Rainbow Blvd, Kansas City, KS 66160 USA; 4 Department of Cancer Biology, University of Kansas Cancer Center, University of Kansas Medical Center, 3901 Rainbow Blvd, Kansas City, KS 66160 USA

**Keywords:** Extensive-stage small cell lung cancer, Immunotherapy, PD-1, PD-L1, Radiation therapy, CTLA-4, CD80, CD28, TIGIT, CD155 (PVR)

## Abstract

The life expectancy of extensive-stage small cell lung (ES-SCLC) cancer patients has not improved in the last 2–3 decades until two recent trials (CASPIAN and IMpower133) showing the addition of anti-programmed death ligand (PD-L1) therapy to chemotherapy has survival benefit over chemotherapy alone. However, such benefit is relatively small and was not even observed in some other trials using immunotherapy, raising the question of optimal chemoimmunotherapy combination in the 1st-line setting for ES-SCLC. Here, we discussed several thought-provoking questions with the focus on IMpower133 and CASPIAN trials.

## To the Editor,

The life expectancy of extensive-stage small cell lung cancer (ES-SCLC) patients has not improved in the last 2–3 decades until two recent trials (CASPIAN [1] and IMpower133 [2]) showing the addition of anti-programmed death ligand (PD-L1) therapy to chemotherapy has survival benefit over chemotherapy alone. However, such benefit is relatively small and was not even observed in some other immunotherapy trials, e.g., CA184-156 study using anti-cytotoxic T cell lymphocyte antigen-4 (CTLA-4) agent ipilimumab [3], raising the question of optimal chemoimmunotherapy combination in the 1st-line setting for ES-SCLC.

Both IMpower133 and CASPIAN were multi-center, phase III randomized studies that reached their primary endpoint of overall survival (OS) benefit. In both studies, the median OS (mOS) was significantly longer in the immunotherapy plus platinum-etoposide group compared to the platinum-etoposide alone group (CASPIAN: 13 [95% CI 11.5–14.8] vs. 10.3 [95% CI 9.3–11.2] months; IMpower133: 12.3 [95% CI 10.8–15.9] vs. 10.3 [95% CI 9.3–11.3] months) (Table 1). Similarly, the progression-free survival (PFS) benefit was observed. The IMpower133 demonstrated that the median PFS (mPFS) was longer in the combined therapy arm (5.2 months [95% CI 4.4–5.6]) compared to the chemotherapy alone arm (4.3 months [95% CI 4.2–4.5]). In the CASPIAN trial, although the mPFS was insignificant, the 1-year progression-free survival rate in the combined treatment group (18% [95% CI 13.1–22.5]) was much higher over the chemotherapy alone group (5% [95% CI 2.4–8.0]), suggesting additional follow-up time for patient events is necessary. It is interesting to notice that IMpower133 reported higher immune-related adverse events (irAEs; Table 1). One reason for this may be reflected in the trial design: while IMpower133 was a double-blinded study, CASPIAN was an open-label trial, which could affect how patients and/or clinicians attribute irAEs.

**Table 1:** A summary of therapeutic efficacy and adverse events

	A vs. B	
	IMpower133	CASPIAN
Efficacy		
PFS (in months)	5.2 vs. 4.3	5.1 vs. 5.4
HR (95% CI)	0.77 (0.62–0.96)	0.78 (0.65–0.94)
PFS%		
At 6 months	30.9% vs. 22.4%	45% vs. 46%
At 12 months	12.6% vs. 5.4%	18% vs. 5%
OS (in months)	12.3 vs. 10.3	13.0 vs. 10.3
HR (95% CI)	0.76 (0.60–0.95)	0.73 (0.59–0.91)
OS% at 12 months	51.7% vs. 38.2%	54% vs. 40%
trAEs		
All grades	94.9% vs. 92.3%	89% vs. 90%
Grades 3–4	56.6% vs. 56.1%	46% vs. 52%
SAEs	22.7% vs. 18.9%	13% vs. 19%
irAEs		
All grades	39.9% vs. 24.5%	20% vs. 3%
Grades 3–4	9.1%* vs. 2.6%*	5% vs. < 1%

*A* chemotherapy + immunotherapy, *B* chemotherapy alone, *HR* hazard ratio, *95% CI* 95% confidence interval, *trAEs* treatment-related adverse events, *AEs* adverse events, *SAEs* severe adverse events, *irAEs* immune-related adverse events

*Calculated using Table S10 in the supplementary appendix provided by the authors

Despite the survival benefit observed in both studies, the absolute improvement in OS remains quite small, and not even statistically significant in the recent KEYNOTE-604 using anti-PD-1 agent pembrolizuamb in combination with platinum-etoposide (not yet published, from Merck’s press release [4]). This is in sharp contrast to the significant OS benefit using anti-PD-1/L1 agents in NSCLC patients [5], suggesting the PD-1/L1 axis may not be the major T cell co-inhibitory pathway, which is consistent with low PD-L1 expression reported in SCLC [6, 7], and co-suppression of other immune checkpoints is likely needed to exert the maximal benefit from immunotherapy. In fact, two recent studies have demonstrated that PD-L1 can bind in *cis* (same cell) to CD80 [8, 9], which interact with both the co-inhibitory receptor CTLA-4 and co-stimulatory receptor CD28. By disrupting PD-L1:CD80 heterodimers, anti-PD-L1 could license high-avidity CD80:CTLA-4 interactions to unleash regulatory T cell-mediated depletion of CD80 from antigen-presenting cells, thereby inhibiting CD28 co-stimulation—this rationalizes the combination of anti-PD-L1 with anti-CTLA-4 for a maximal anti-tumor effect [9]. In consistent with this, CASPIAN has a 4-drug arm including the anti-CTLA-4 agent tremelimumab (in addition to durvalumab plus platinum-etoposide) that is currently ongoing. Comparison of this arm to the other two (platinum-etoposide with or without durvalumab) will be highly anticipated despite the earlier negative result from the CA184-156 study [3]. Furthermore, co-targeting other co-inhibitory receptors such as the T cell immunoreceptor with Ig and ITIM domains (TIGIT) is also of great interest (there is an ongoing study SKYSCRAPER-02, ClinicalTrials.gov Identifier: NCT04256421), especially considering its ligand CD155 (or poliovirus receptor (PVR)) is broadly expressed in both the SCLC cell lines and patient tumor tissue [10], and co-blockade of TIGIT and PD-1/L1 was found synergistic [11]. Finally, consolidative thoracic radiotherapy (CTRT) may further improve the survival benefit since 75% of patients with ES-SLCC could have persistent intrathoracic disease following induction chemotherapy [12], and CTRT has been shown to provide an OS benefit in patients who respond to initial chemotherapy [13]. It is hoped that radiation could enhance the immunogenicity of these tumors through promoting the release of tumor antigens [14], therefore enhance immunotherapy response. Importantly, a recent phase 1 trial of pembrolizumab in combination with thoracic radiation after induction chemotherapy for ES-SCLC demonstrated this combination was well tolerated [15].

In summary, these two studies provided strong evidence to support the use of immune checkpoint blockade in ES-SCLC. However, questions remain regarding whether anti-PD-1/L1 in combination with other immune checkpoint inhibitors could further enhance the overall survival, and whether radiotherapy should be combined with chemoimmunotherapy in ES-SCLC.

## Data Availability

Extracted from the literature.

## Abbreviations

APC: Antigen-presenting cell
CTRT: Consolidative thoracic radiotherapy
HR: Hazard ratio
NSCLC: Non-small cell lung cancer
OS: Overall survival
PCI: Prophylactic cranial irradiation
PD-1: Programmed cell death protein 1
PD-L1: Programmed death-ligand 1
PFS: Progression-free survival
SCLC: Small cell lung cancer
